# Triceps Surae Ia Proprioceptive Weighting in Postural Control During Quiet Stance with Vision Occlusion

**DOI:** 10.3390/jfmk10040430

**Published:** 2025-11-05

**Authors:** Gordon R. Chalmers

**Affiliations:** Kinesiology and Physical Education Program, Department of Health and Human Development, Western Washington University, Bellingham, DC 98225, USA; chalmers@wwu.edu

**Keywords:** nervous system, proprioception, mechanoreceptors, postural control, balance, vision, human, vibration, triceps surae, muscle

## Abstract

**Background:** Visual, vestibular, proprioceptive and cutaneous sensory information is important for postural control during quiet stance. When the reliability of one source of sensory information used to detect self-motion for postural control is reduced, there may be a reweighting of inputs within and/or across the remaining sensory systems determining self-motion for postural control. Muscle vibration, which creates an illusion of muscle stretch and a compensatory movement to shorten the vibrated muscle, may be used to determine the weighting of muscle spindle Ia proprioception in postural control. The objective of this study was to determine the effect of vision occlusion on triceps surae (TS) Ia proprioceptive weighting in postural control during quiet stance, utilizing an 80 Hz muscle vibration stimulus and a quantitative measure of the body’s anterior to posterior ground center of pressure (COP) response to TS muscle vibration in subjects standing freely. **Methods:** Subjects (N = 41; mean (standard deviation), 19.6(2.0) years) were examined as they stood with eyes open (EO) or eyes closed (EC). Ground COP was measured during quiet standing with and without bilateral vibration of the TS muscles. **Results:** The mean backward COP shift induced by TS vibration was significantly greater during the EC condition compared to EO (EC: −4.93(1.62) centimeters; EO: −3.21(1.33) centimeters; *p* = 6.85 × 10^−10^; Cohen’s d = 1.29). Thirty-seven subjects increased, and two subjects decreased their vibration-induced COP backward shift in the EC condition compared to EO, although the magnitude of the change varied. **Conclusions:** The results support the idea that, for most young subjects, there is an increased triceps surae Ia proprioceptive weighting for postural control during EC stance, possibly due to the need for postural control to depend more on non-visual feedback.

## 1. Introduction

Visual, vestibular, proprioceptive and cutaneous sensory information is important for the central control of steady upright standing (termed postural control in this study) during quiet stance [1,2,3,4]. The ankle is an important source of the proprioceptive information used for postural control [4,5]. For ankle kinesthetic sense, muscle spindles serve as the major source of information, with skin receptors also playing a role [4,6,7,8]. When the reliability of one source of sensory information used to detect self-motion for postural control is reduced, there may be a reweighting of that and other sensory inputs utilized for postural control [9,10,11]. For example, when the ability of ankle proprioception to predict body position during stance is reduced by standing on an unstable surface, causing the Ia signals from ankle muscles to be an unreliable indicator of body sway due to surface movements, there is a down weighting of the effect of the triceps surae (TS) Ia signal. Simultaneously, an upweighting of the effect on postural control of the lumbar paraspinal musculature Ia signal [12,13] and of the vestibular system [14] may occur.

Although multiple studies have demonstrated that the weighting of ankle proprioceptive input can be acutely decreased when needed to aid postural control [12,13,15,16], none have clearly demonstrated that it can be increased when visual information is absent. An increased ankle weighting could be beneficial for postural control when visual information is not available, such as when balancing with eyes closed (EC) is practiced for rehabilitation [17,18,19] or exercise training [20].

Muscle vibration may be used to determine the weighting of muscle proprioception for postural control during quiet standing [12,13,16,21]. Muscle vibration causes an increase in the discharge rate of spindle primary (type Ia) endings and the illusion that the vibrated muscle is being stretched [22]. During quiet stance, this illusion in the supporting muscles leads to a compensatory shift of the body’s center of mass (COM) and ground center of pressure (COP) in the direction that shortens the vibrated muscle. For example, when standing, TS muscle vibration results in an illusion of forward leaning, with a resulting posterior shift of the COP [12,13,16,21,23].

Only two studies have examined the proprioceptive weighting of the ankle muscle system for postural control during quiet stance with eyes open (EO) versus a condition lacking visual information. Both studies have methodological limitations, and report contradictory results. Lackner and Levine [24] found that during an EO condition in an illuminated room that a TS muscle vibratory illusion was not reported by subjects, but the vibratory illusion did occur in a dark room. These results suggest a greater weighting of the TS muscle Ia proprioceptive system on postural control when visual information is absent, compared to an EO condition. Lackner and Levine [24], however, relied on qualitative subjective reports by the subjects of a body movement illusion occurring with vibration while they were fixed to a backboard rather than standing freely. In contrast, Toosizadeh and colleagues examined the anterior–posterior (AP) COM position in subjects, finding a shift in COM during an EO but not during an EC condition with gastrocnemius muscle vibration [25]. This may demonstrate a lower weighting of gastrocnemius muscle Ia proprioception for postural control during an EC condition, compared to EO. However, the 40 hertz (Hz) vibration frequency used by Toosizadeh and colleagues was not optimal for examining Ia proprioceptive weighting because spindle stimulation reduces as vibration frequency is reduced [2]. Forty Hz is only slightly above the approximately 30 Hz threshold for eliciting vibration-induced postural responses in ankle muscles in healthy adults [23] and is much lower than the approximately 80 Hz vibration frequency that is optimal to stimulate spindle primary endings [6,26]. Further, the lack of a vibratory-induced sway in the EC condition reported by Toosizadeh and colleagues may have been due to a rise in the threshold for discriminating vibratory stimuli, which may occur with eye closure [25,27], revealed by their use of a stimulation frequency only slightly above the threshold, as opposed to a change in the weighting of the Ia proprioceptive system.

The purpose of this study was to determine the effect of vision occlusion on TS Ia proprioceptive weighting for postural control during quiet stance. The unique features of this study were that it was the first examining this question using an 80 Hz muscle vibration stimulus and a quantitative measure of the body’s COP response to muscle vibration in subjects standing freely. Eye closure eliminates the contribution of visual sensory cues to postural control. It has been suggested that, while in the EC condition, compared to EO, the need for postural control to depend more on non-visual feedback would result in a greater weighting of TS Ia proprioceptive information. This increased weighting would be measured by a greater shift in the body’s AP COP being induced by TS muscle vibration in the EC compared to the EO condition. Accordingly, the research hypothesis was that during quiet stance, the mean AP COP shift in the direction of the bilaterally vibrated TS muscles would be greater with vision occlusion compared to EO.

## 2. Materials and Methods

### 2.1. Participants

A priori, a G*power calculation (version 3.1.9.7) determined that 34 subjects were needed to detect a moderate treatment effect (0.5) with an alpha error probability of 0.05 and a statistical power of 0.8 for the statistical analysis used (discussed below) [28]. Subjects aged 18 to 40 years with no neurological, vestibular, visual or lower limb musculoskeletal disorders or neuromuscular medications were recruited. Visual corrective lenses were allowed. This study was conducted in accordance with the Declaration of Helsinki, and approved by the Institutional Review Board of Western Washington University (protocol code 9489EP23, approved on 25 January 2024). Prior to testing, informed consent was obtained from all subjects involved in this study by having them read and then sign a written consent form.

### 2.2. Experimental Procedures and Data Acquisition

Subjects, wearing shorts, stood barefoot on a force plate (Balance Tracking Systems, San Diego, CA, USA) with arms loosely hanging at their sides and feet shoulder-width apart and a self-selected splay angle. Subjects stood straight and relaxed without moving, in a quiet room, with no distractions. Foot position was marked on the plate surface to minimize change if repositioning was required.

**Figure 1:**
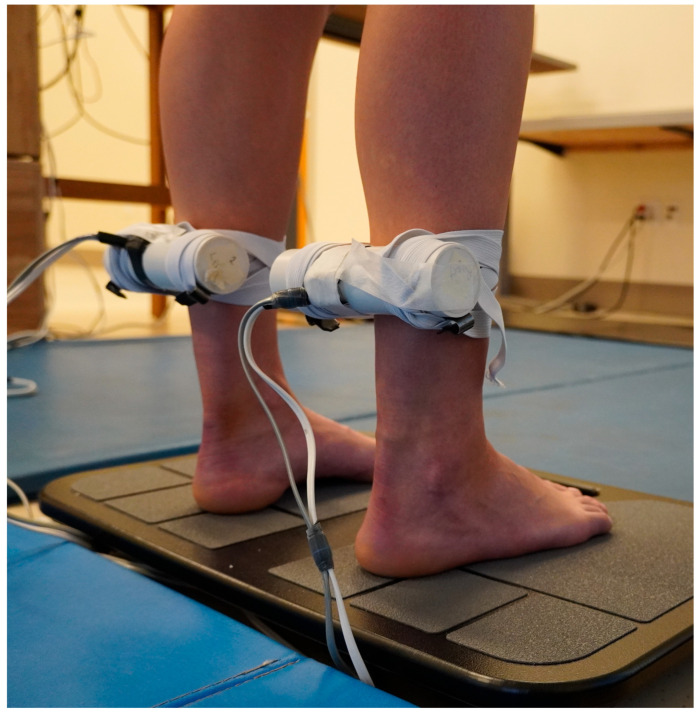
Vibrators were attached bilaterally to the triceps surae muscle.

Vibrators, 15 cm long, 3.8 cm in diameter (BestTong, China https://www.besttong.co/, A00000531), equipped with an accelerometer (Dimension Engineering, Hudson, OH, USA, DE-ACCM6G), were attached bilaterally to the TS muscle. Elastic straps limited vibration of nearby tissues [29] (Figure 1). After attachment, each vibrator was calibrated to vibrate at 80 Hz (digitized at 10,000 Hz, Micro 1401, Spike2 software, version 7.17, Cambridge Electronic Designs, Milton, UK). All vibration stimuli were simultaneously applied bilaterally. Subjects then received two seconds of vibration to experience it and minimize startle response at trial onset. To prepare subjects for possible unexpected sensations, they were instructed that the vibrators would vibrate their muscles, that they may perceive a shift in their body position, and that they were to let the body automatically handle its balance. They were instructed not to interfere with the sensations experienced, not to resist any body tilt, and a researcher was positioned nearby to prevent a fall if needed. Padded floor mats were placed around them for safety. Briefing subjects on what to expect during the experiment increases safety and the likelihood of them experiencing the vibration movement illusion [26,30]. However, the instructions did not specify the direction to prevent intentional responses by subjects. Subjects were randomly assigned to the testing sequence of EO, followed EC, or the reverse. For each trial, the subject adopted the condition for 20 s, to allow time for adaptation to the new sensory condition [31,32], then data collection from the force plate was initiated. Twenty seconds later, vibration was initiated and continued for 20 s. During the 40 s period BTrackS™ Assess Balance software (version 7.5.6, Balance Tracking Systems, San Diego, CA, USA) recorded the subject’s COP on the force plate at 25 Hz. During EO trials, subjects gazed straight ahead at a self-selected spot in a poster positioned at eye level, positioned 300 cm away. A visual target was employed to decrease standing sway during the EO test [33] and enhance the difference between the EO and EC conditions, compared to staring at a blank wall. The EC condition was produced by the subject closing their eyes, which was monitored. Diffused room lighting from above ensured that no source of light information could provide motion information during EC testing. In both the EO and EC conditions, the head and eyes were held in a forward-facing position to minimize possible effects of head or eye movement on posture [34,35]. If during a trial a subject moved more than the typical illusion-induced body sway, the trial was rejected and repeated once after a 5 min seated rest period. To minimize a possible post-vibration carryover effects between trials [36], subjects had five minutes of seated rest between trials [25,27], and each trial condition of EO and EC was conducted only once. The use of a single trial for each condition is common in muscle vibratory studies [12,13,25,27,37,38,39]. To minimize muscle thixotrophy effects, subjects stood from a seated resting position immediately before each trial, moving directly onto the force plate, to ensure a similar muscle contraction history across both conditions [40]. To demonstrate that the vibration frequency did not change once set, the above procedures were repeated six times in each of two subjects with EO during both trials. The frequency of the vibrators were recorded during setup, the first trial and the second trial, for each of the right (R) and left (L) legs. The COP data from these trials for these two subjects was not recorded.

### 2.3. Data Analysis for Each Subject

COP data were filtered using a 2nd-order zero-lag 4 Hz low-pass Butterworth filter in BTrackS™ Assess Balance software, then exported to an Excel file (version 2411, Microsoft Office 365, Redmond, WA, USA). TS muscle vibration produces almost exclusively AP body movements [41], so only AP responses were analyzed for the COP measures [2,12,13,42]. The mean AP COP was determined for seconds 0–15 during the non-vibration period and seconds 25–40 during the vibration period. The first 5 s of the vibration period were omitted because in some subjects the vibration illusion can take several seconds to occur [16,24,43]. For the EO and EC conditions, the AP COP displacement induced by vibration was calculated using Equation (1):

(1) (mean AP COP during vibration − mean AP COP during no vibration)

A positive value indicates forward movement, and a negative value indicates backward movement, so larger negative difference values indicate greater backward lean induced by vibration [12,13,21,37]. The greater the magnitude of the vibration-induced shift in the standing body’s COP during muscle vibration, the greater the weighting of the vibrated muscle’s Ia proprioceptive signal for postural control [12,16,21,37].

### 2.4. Statistical Analyses

To determine if a subject responded to the vibration stimulus with a vibration-induced shift in the AP COP, Cohen’s d was calculated to compare the mean AP COP during the EO non-vibration and vibration conditions for the subject. Cohen’s d values meeting or exceeding, 0.2, 0.5, and 0.8, indicated small, medium, and large effects [44]. Only subjects who responded to the vibration stimulus during the EO condition with at least a small backwards vibration-induced shift in their AP COP were included in the subsequent data analysis. Data were tested for deviations from normality using the Shapiro–Wilk test. To determine the effect of eye closure on TS muscle Ia proprioceptive weighting, a repeated measures two-tailed *t*-test, and a Cohen’s d calculation, compared the mean AP COP displacement-induced by vibration during the EO and EC conditions. Differences in the magnitude of the vibration-induced shift in a body’s COP across testing conditions indicate differences in the weighting of the vibrated muscle’s Ia proprioceptive signal for standing postural control [12,16,21]. For the tests of the vibrator frequency consistency across trials, the frequency data were analyzed using a two-way between (right and left legs) within (set-up, trial 1, trial 2) ANOVA. SPSS (version 29.0.2.0, IBM Corporation, Armonk, NY, USA) and Microsoft Excel were used for calculations. Significance level was set at *p* < 0.05, and the results are reported as mean (standard deviation).

## 3. Results

**Figure 2:**
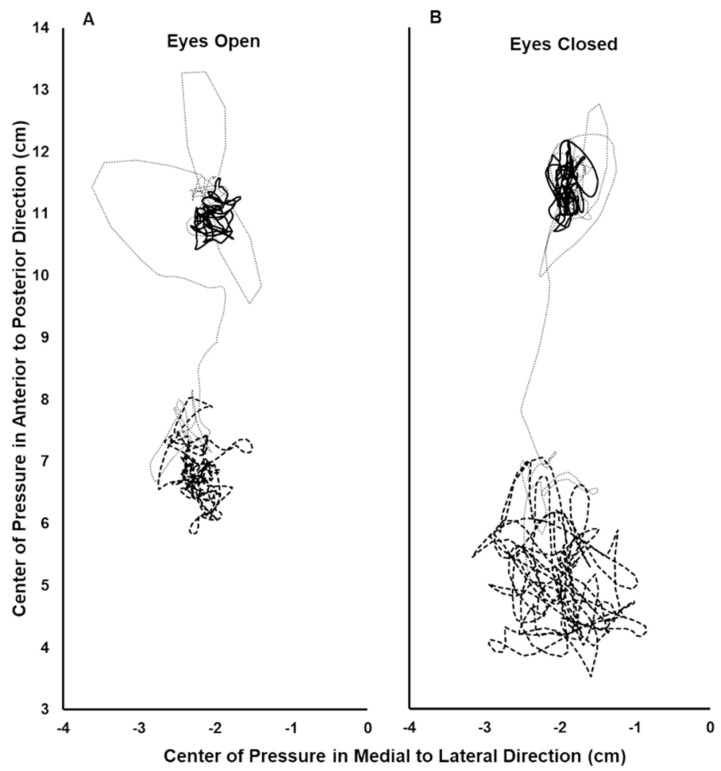
Center of pressure (COP) record during the eyes open (**A**) and eyes closed (**B**) trials for a single subject. The COP path during the initial 15 s period analyzed with no vibration (solid line), the final 15 s period analyzed with vibration (thick dashed line), and the 10 s interval between these periods (thin dotted line). In the anterior–posterior direction, greater positive values indicate a more anterior position.

**Figure 3:**
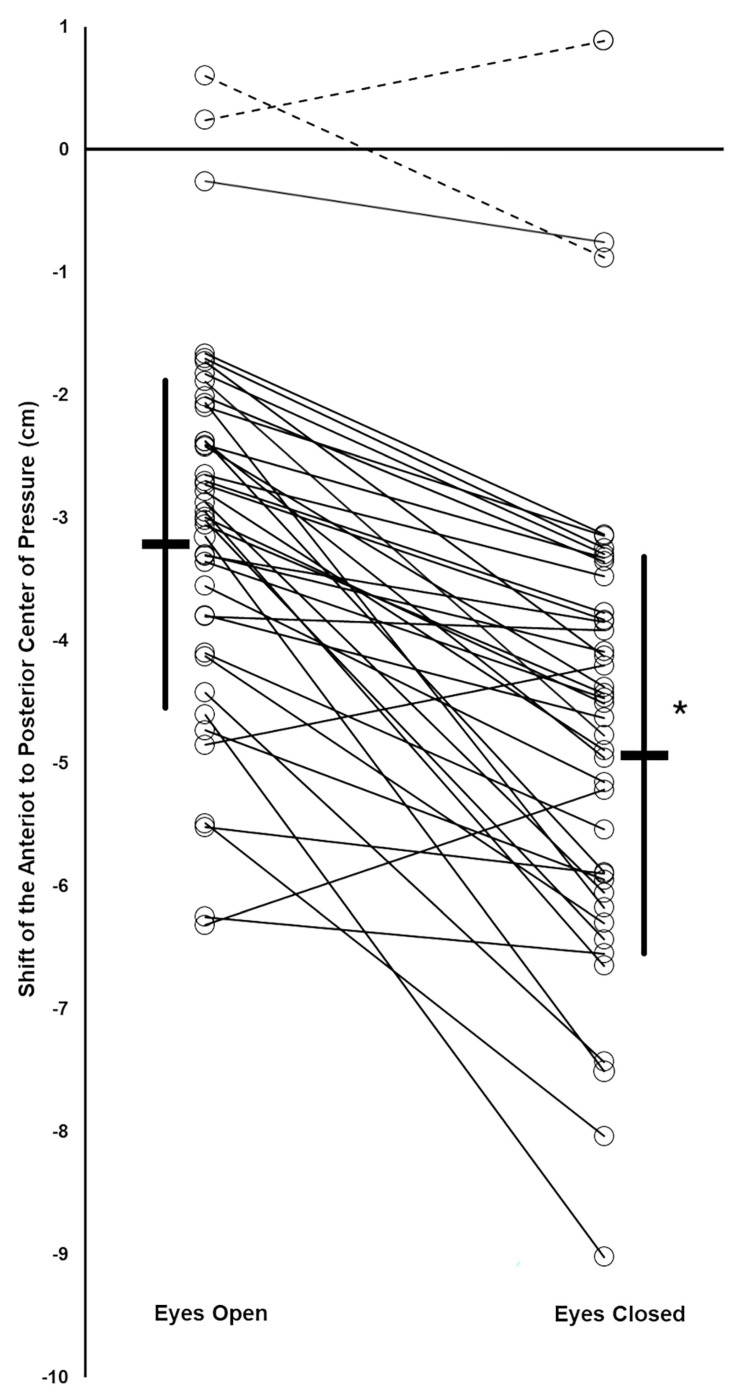
Vibration induced a shift in the anterior–posterior (AP) center of pressure (COP) of the subjects. Group mean values (thick horizontal bars) with standard deviation (thick vertical lines). For each subject, their AP COP shifts during EO and EC conditions are shown by open circles, with thin lines connecting the EO and EC data for the same subject (thin solid lines: subjects who responded to the muscle vibration during the EO condition; thin dotted lines: subjects who did not respond to the muscle vibration during the EO condition). * Significant difference between EO and EC condition means.

Forty-one subjects were studied (age: 19.6(2.0) years; weight: 147(30) lbs; self reported as female = 25, male = 15, not reported = 1). Two subjects did not respond to the vibration stimulus in the EO condition with at least a small backward shift of their AP COP, based on Cohen’s d criterion. The remaining 39 subjects were included in the data analysis. Thirty-eight of these subjects had a large backward vibration-induced AP COP shift and one had a medium shift during the EO condition, based on Cohen’s d criteria. An example of a subject’s COP record during the EO and EC trials is shown in Figure 2. The data did not deviate from normality. The mean backward COP shift induced by TS vibration was significantly greater during the EC condition compared to EO, and the difference was large, based on Cohen’s d criterion (EC: −4.93(1.62) cm; EO: −3.21(1.33) cm; *p* = 6.85 × 10^−10^; Cohen’s d = 1.29) (Figure 3). Thirty-seven of the 39 subjects increased their vibration- induced AP COP backward shift in the EC condition, compared to EO. The magnitude of the increase varied, however, as reflected by the range of downward slopes of the thin solid lines in Figure 3. In contrast, two subjects decreased their vibration-induced AP COP backward shift in the EC condition, compared to EO, as shown by the two upward-sloping thin solid lines in Figure 3. One subject who did not respond to the vibration during the EO condition with a backward shift of their AP COP did demonstrate a large backward shift during the EC condition, based on Cohen’s d criteria (thin downward-sloping dashed line, Figure 3). This subject also increased their TS muscle Ia proprioceptive weighting with EC, although they could not be included in the calculation of the group means because they did not respond to the vibration during the EO test.

Tests of vibrator frequency consistency across the trials there showed no significant change in vibration frequency over the course of the experiment or when comparing the two legs (setup: R = 79.5(0.4) Hz, L = 79.5(0.4) Hz; trial 1: R = 79.4(0.7) Hz, L = 79.6(0.5) Hz; trial 2: R = 79.4(0.5) Hz, L = 79.6(0.5) Hz, interaction *p* = 0.45, trial main effect *p* = 0.96, leg main effect *p* = 0.36).

## 4. Discussion

The present results are the first to demonstrate that ankle Ia proprioception can be acutely upweighted when visual information is not available for postural control, and that individuals differ greatly in the magnitude of this change (Figure 3). This study was unique because it examined this question utilizing an 80 Hz muscle vibration stimulus, optimal for stimulation of muscle spindles, and employed a quantitative measure of the body’s COP response to muscle vibration in subjects standing freely.

A direct comparison of the magnitude of the increased TS Ia proprioceptive weighting with eye closure reported in the current study and the similar finding by Lackner and Levine is not possible. The latter relied on qualitative reports of illusory body movement by subjects who were not freely standing [24]. Toosizadeh and colleagues found opposite results in similar healthy young subjects [25]. They reported a vibration-induced shift in COM during the EO, but not during the EC condition, possibly indicating a greater weighting of gastrocnemius muscle Ia proprioception for postural control with EO [25]. Toosizadeh and colleagues, however, utilized a 40 Hz muscle vibration frequency that was only slightly above the threshold for eliciting vibration-induced postural responses in soleus and tibialis anterior (TA) muscles [23] and therefore may not be optimal for examining the research question of the present study. Additionally, the lack of a vibration effect on posture during the EC condition, which as they observed may have been due to a rise in threshold for discrimination of vibratory stimuli occurring with eye closure [25,27], as opposed to a lower Ia proprioceptive weighting.

We hypothesizing that an eye closure-induced change in ankle proprioceptive weighting for postural control is reasonable due to the importance of proprioception, particularly from the ankle, for postural control [4,5,41]. The selection of the ankle plantar flexor muscles as targets for measuring ankle proprioceptive feedback during eye closure is appropriate because standing sway increases under EC conditions [45,46], with AP sway, the primary direction influenced by the TS muscles being tested, increasing more than medial lateral sway [45,46,47,48,49]. Future studies examining TA muscle Ia proprioceptive weighting with eye closure would be interesting, given the possibility that the TA muscles may be even more important than the TS muscles for providing AP proprioceptive feedback from the ankle [4,50]. The mechanism by which TS muscle Ia proprioceptive weighting was changed in subjects during EC stance was not examined. It is likely that it was not due to a change in muscle spindle sensitivity in the TS muscles because eye closure during stance does not change the fusimotor control of pre-tibial muscles [51]. Therefore, an alteration in Ia proprioceptive information central processing likely occurs [21].

The amount of backward lean induced by TS muscle vibration with EO varied widely between subjects, including two subjects with no vibration-induced body movement, as has been previously reported [16,24]. A novel finding in this report is the wide variability in the increase in TS Ia proprioceptive weighting with eye closure, and that two subjects instead showed a decrease in this weighting with eye closure, as indicated by the variation in slopes of the thin solid lines in Figure 3. Isableu and colleagues have previously established that individuals vary in their degree of dependence on the visual field for body postural control [52]. Individuals classified as highly visual-field-independent subjects were better able to use nonvisual information from proprioceptive and vestibular sources for postural control when challenged with reduced visual cues of self-orientation and self-motion [52]. In contrast, subjects with high visual field dependence exhibited difficulty utilizing these nonvisual cues for postural control [52]. Accordingly, intersubject differences in visual field dependence and abilities to use proprioceptive sources for postural control may explain the varying degrees and directions of change in TS Ia proprioceptive weighting when vision was removed in the present study.

The results of this study have good ecological validity for rehabilitation clinics [17,18,19] or exercise facilities [20] where individuals practice standing balance with their eyes closed while freely standing, similar to the EC condition tested. Based on the present results showing that TS Ia proprioceptive weighting can be acutely increased during EC standing, it would be interesting to determine if it also increases following ankle proprioceptive training aimed at enhancing joint position sense [53,54], and in contexts where EC [55] and vision disruption [56,57] balance training have been demonstrated to be superior to EO balance training. The eye closure employed in the present study is not the equivalent of a dark room with EO. In a dark room with eyelids open there are smaller displacements for a subject’s vertical projection of the center of gravity and for the difference between the COP and the vertical projection of the center of gravity, compared to when standing with eyelids closed [49]. The present results may, therefore, not have ecological validity to dark conditions with open eyes.

Certain limitations to this study should be acknowledged. The vibration applied was intended to stimulate ankle TS spindle Ia proprioceptive feedback. However, cutaneous stimulation of the leg also affects proprioception from the ankle joint [7,8]. Cutaneous stimulation would be affected by the straps holding the muscle vibrators and the vibration employed. However, cutaneous stimulation of the leg would have been the same during both the EO and the EC trials, and so would not be the cause of the difference in mean AP COP backward shift with vibration when comparing the EC and EO conditions. The current study utilized the test measurement of vibration-induced backward body sway. However, backward body sway changes plantar foot pressure distribution, and plantar somatosensory feedback also contributes to postural control [7,58]. Accordingly, a change in plantar somatosensory feedback during the vibration testing procedure may have affected the degree of backward sway measured during both the EO and EC conditions. Eye movement during EO conditions affects postural control [34,35], but this was minimized or eliminated by having the subjects focus on one visual target during EO testing, and observations of the subjects. It is unlikely that any eye movement during EC testing would affect the results, because saccades when the eyes are closed do not affect postural sway [59]. This study included only young healthy adults as subjects. It is unknown whether the results would apply to individuals outside of this population group.

## Data Availability

The data presented in this study are openly available in Harvard Dataverse at https://doi.org/10.7910/DVN/JJS2FK.
